# A Benign Renal Tumor With Serious Consequences: A Case Report of Juxtaglomerular Cell Tumor and Chronic Renal Disease in a Teenager

**DOI:** 10.1155/crpe/9318115

**Published:** 2025-06-13

**Authors:** Rachael Courtney, Erin Dahlinghaus, Abiodun Omoloja, Jeffrey T. Boyd, Michelle Smith, Laura Biederman, Israel Ndengabaganizi, Daniel Keith Robie, Ahmad Al Dughiem

**Affiliations:** ^1^Departments of Pediatrics, Dayton Children's Hospital and Wright State University Boonshoft School of Medicine, Dayton, Ohio, USA; ^2^Department of Pathology, Dayton Children's Hospital and Wright State University Boonshoft School of Medicine, Dayton, Ohio, USA; ^3^Department of Pathology, The Ohio State University, Columbus, Ohio, USA; ^4^Department of Surgery, Dayton Children's Hospital and Wright State University Boonshoft School of Medicine, Dayton, Ohio, USA

## Abstract

Juxtaglomerular cell tumor (JGCT), or reninoma, is a rare benign renal neoplasm. It is characterized by severe hypertension and hypokalemia due to excessive renin produced from the juxtaglomerular apparatus on the afferent arteriole of the glomerulus. Herein, we report a case of JGCT in a 15-year-old female who presented with severe hypertension. She was found to have elevated levels of renin and aldosterone with hypokalemia and she responded to angiotensin-converting enzyme inhibitors (ACEi). Abdominal MRI demonstrated a 4-cm left renal tumor. A radical nephrectomy was performed, and histology showed a well-circumscribed tumor consisting of sheets of polygonal to ovoid cells consistent with a JGCT. After surgery, the renin and aldosterone levels normalized, and blood pressure was controlled with small dose of ACEi medication. Unfortunately, the patient remained in Stage III chronic kidney failure due to the longstanding damage of uncontrolled hypertension prior to the diagnosis. We hereby review the literature and discuss the differential diagnosis.

## 1. Introduction

Juxtaglomerular cell tumor (JGCT), first described in 1967, is a rare benign tumor of the kidney affecting mostly adolescent females [1]. It is characterized by severe hypertension with hypokalemia due to excessive renin production. Unfortunately, delayed diagnosis can lead to chronic kidney damage even in the unaffected renal tissue from longstanding hypertension.

Surgical removal of the tumor is the primary treatment of choice [2]. However, while the vast majority of patients have benign disease and can be managed effectively with a partial nephrectomy, there have been case reports of malignant transformation and even metastasis necessitating additional treatment [3–6].

Considering the extensive differential diagnoses for hypertension and the rarity of JGCT, the diagnosing physician must have a high index of suspicion for JGCT. Early recognition and management can help prevent cardiovascular and renal morbidity and mortality, improve quality of life, and limit socioeconomic liabilities [7].

## 2. Case Presentation

A 15-year-old previously healthy girl presented to her primary care provider for an annual visit where she was found to have a high blood pressure (BP) of 180/110 mmHg. Aside from a history of an occasional headache, there were no clinical symptoms. She denied nausea, vomiting, syncope, or visual disturbances. There were no family members diagnosed with hypertension, renal disease, or cardiovascular disease.

The patient was referred to our tertiary children's hospital where hypertension was confirmed by BP readings of 180–185 mmHg systolic and 110–120 mmHg diastolic in all extremities. Pulses were palpable in all limbs with no radiofemoral delay. Examination of the heart revealed normal heart sounds and no murmurs. There was no organomegaly or masses palpable in the abdomen. Examination of the neck revealed no bruit or thyroid gland enlargement. Neurological examination was normal.

Echocardiography was performed to evaluate for hypertensive target organ damage and revealed moderate to severe concentric left ventricular hypertrophy and elevated left ventricular mass index of 46 gm/m2.7 (mean value = 40 g/m2.7).

Basic laboratory investigations showed normal urinalysis without blood and protein, normal complete blood counts, and normal liver function. However, she did have an elevated serum creatinine of 1.13 mg/dL (reference range 0.7–1.1 mg/dL), hypokalemia of 2.8 mmol/L (3.8–5.1 mmol/L), and elevated serum CO_2_ of 33 mmol/L (16–25 mmol/L). Her cystatin C was 1.1 mg/L and calculated glomerular filtration rate was 56 mL/min/1.73 m^2^ (by CKiD U25 eGFR equation using creatinine and cystatin C), placing her in Stage 3 chronic renal failure (defined by a GFR between 30 and 59 mL/min/1.73 m^2^). Over the next few days, testing of vanillylmandelic acid was normal at 4 mg/g (0–9 mg/g) and plasma metanephrine was normal at 0.3 mmol/L (0–0.49 mmol/L).

**Figure 1:**
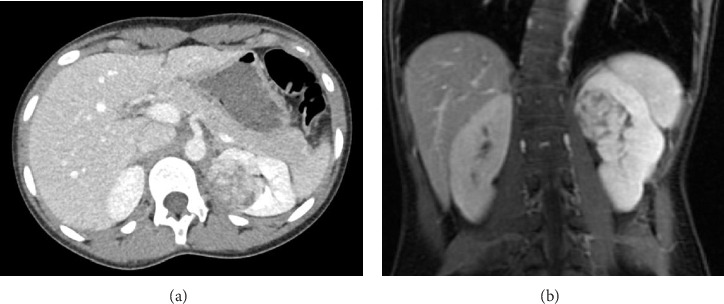
A mass in the upper pole of the left kidney shown by CT imaging (a). MRI imaging (b) showed a heterogeneous mass within the upper pole of the left kidney measuring 3.8 × 4.1 × 3.6 cm. The lesion demonstrated intermediate to high signal on T2-weighted imaging, intermediate to low signal on T1-weighted imaging, and enhancement similar to the kidney with multiple irregular areas of decreased signal present throughout the lesion. No other focal renal lesions, no hydronephrosis, and no ureteral dilation were noted, and the inferior vena cava was patent.

Because of the association of severe hypertension and hypokalemia, the subsequent work-up focused on the causes of renin-mediated hypertension. Plasma renin and aldosterone levels were markedly elevated at 68.4 ng/mL/hr (0.5–3.3 ng/mL/hr) and 24.8 ng/dL (< 16.0 ng/dL), respectively. A renal ultrasound revealed a rounded area of increased echogenicity in the upper pole of the left kidney. Further imaging was done including CT and MRI imaging which confirmed nearly a 4-cm, heterogeneous mass within the upper pole of the left kidney (Figure 1).

The differential diagnosis of renal tumors in an adolescent is a bit different than that in younger children. Renal cell carcinomas represent two-thirds of all renal tumors in patients aged 15–19 years. This is followed by nephroblastoma and then a variety of rare malignant tumors including rhabdoid tumor, clear cell carcinoma, and even benign tumors such as mesoblastic nephromas. The vast majority of renal tumors at this age are malignant; thus, biopsy is typically contraindicated as entering and breaking the renal capsule can lead to seeding of malignant tumor cells into the abdominal cavity. Furthermore, her tumor abutted the hilum making a partial resection with proper surgical margins near impossible. She completed a staging workup including Fluorine-18 fluorodeoxyglucose positron emission tomography/computed tomography (18F-FDG PET/CT) imaging which revealed that the tumor had moderate PET-avidity with a SUV of 5.2 (SUV or standardized uptake values > 2.5 are considered abnormal). There were no signs of metastatic involvement. Fearing chronic damage in the unaffected kidney due to the longstanding hypertension, we also obtained a nuclear renal scintigraphy study that showed 55% function in the affected left kidney and 45% function in the unaffected right kidney.

Thus, the decision was made to stabilize her hypertension and proceed with a radical nephrectomy as an outpatient. The patient remained clinically well during her stay in the hospital. Her hypertension was treated with a combination of calcium channel blocker nifedipine XL (60 mg/day) and ACEi enalapril 10 mg daily with normalization of BP (120/80) and resolution of headaches. She was stable and discharged for surgical management as an outpatient. Approximately 15 days after her initial presentation, she underwent a left radical nephrectomy with lymph node sampling. There were no surgical complications.

**Figure 2:**
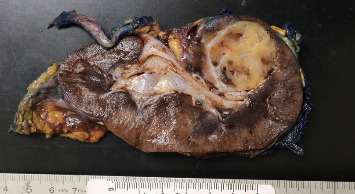
Gross pathology of the left kidney.

**Figure 3:**
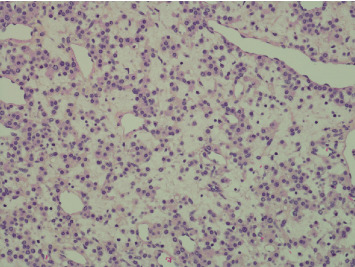
Packed, uniform round-to-polygonal cells.

**Figure 4:**
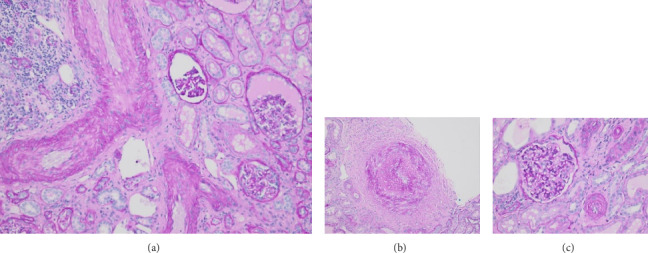
(a) Hypoperfused glomeruli with periglomeruli fibrosis adjacent to a vessel with intimal fibrosis (PAS 10x). (b) Severe intimal fibrosis in a vessel (PAS 10x). (c) Mildly enlarged glomerulus with arteriole showing concentric remodeling (PAS 20x).

**Figure 5:**
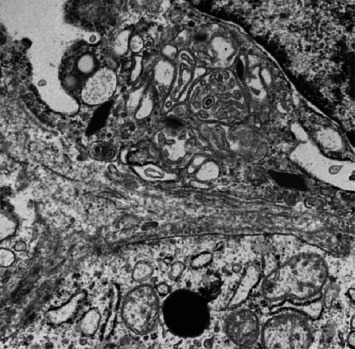
Rhomboid-shaped renin protogranules.

Pathology evaluation noted a well-circumscribed tan nodule measuring 3.7 × 3.0 × 2.9 cm with hemorrhage involving the upper pole of the left kidney and pushing into the renal sinus. The lesion did not extend to the renal capsule or soft tissue margin (Figure 2). Light microscopic examination showed a tumor composed of closely packed, uniform round to polygonal cells with a granular, eosinophilic cytoplasm in addition to secondary changes in arteries and glomeruli (Figures 3 and 4). Immunohistochemical studies showed that the tumor cells were strongly and diffusely positive for CD34. The neoplastic cells were negative for colloidal iron, PAX-8, B-catenin, CD10, CK7, CD117, CA9, and S100. The constellation of histological and immunohistochemical features was consistent with the diagnosis of JGCT (reninoma). The diagnosis was confirmed by electron microscopy revealing the presence of rhomboid dense deposits (renin crystals) in the cytoplasm of the tumor cells (Figure 5).

Three weeks after surgery, plasma renin activity was within normal and BP was controlled with only enalapril 10 mg daily. Unfortunately, she did remain in Stage III chronic kidney disease in the remaining right kidney with an estimated glomerular filtration rate of 46 mL/min/1.73 m^2^ (by CKiD U25 eGFR equation using creatinine and cystatin C). This impairment in renal function is probably explained by the irreversible changes in the kidney tissue secondary to longstanding hypertension as demonstrated on the pathology review (Figure 5).

## 3. Discussion

There are approximately 250–300 reported cases of JGCT in the literature; however, due to their rarity, the true incidence is unknown. In two larger reviews, the majority of cases occurred in the teens and 20s with a median age of 25 years and an approximately 2:1 female predominance [8, 9]. One review noted that patients younger than 20 years had a shorter average time to diagnosis than adults at 2.6 years and 6 years, respectively [9]. The average patient has been found to have a renin value 7.89 times the upper limit of normal [9]. This is consistent with what we saw in our 15-year-old female patient incidentally discovered to have hypertension with only occasional headaches and a renin that was 20-fold the upper limit of normal.

Causes of renin-mediated hypertension include conditions that decrease renal blood flow including renal artery stenosis after trauma or infection, mass effect from any renal tumor onto the vascular flow, renal artery thrombosis, and very rare renin-secreting tumors such as JGCT [10]. Given the initial assumption that our patient's tumor was very likely malignant, it stood to reason that she could have renin-mediated hypertension from anatomical damage to the renal artery abutting the mass. However, with the diagnosis of a JGCT, it is clear that her renin-mediated hypertension occurred through a separate mechanism with renin-mediated vasoconstriction. The renal glomerulus contains juxtaglomerular cells (JGCs) within the afferent arterioles. These cells create prorenin which is cleaved into the proteolytic enzyme renin and released from the kidney in response to decreases in BP. Renin then acts on the angiotensin enzyme and helps form Angiotensin I and then Angiotensin II. Angiotensin II then works through a series of step to cause vasoconstriction leading to increased BP. Since JGCT create masses of additional JGCs, there is an excessive increase in prorenin and eventually vasoconstriction leading to severe hypertension.

Longstanding hypertension leads to end organ damage; thus, treatment must be aimed at resolving the hypertension. While removing the offending renin-producing tumor as the cure, it is often vital to emergently employ BP medications to reduce symptoms and ensure they are stabilized for their upcoming surgical resection. Rapid reduction in BP soon after commencement of nifedipine in our patient seemed to follow other observations that calcium channel blockers are very effective in JCT-induced hypertension [8]. Interestingly, use of angiotensin-converting enzyme inhibitors (ACEi) for BP control in JGCT has been mixed, although we had no difficulty controlling our patient's BP with relatively minimal dose of enalapril [8]. For our patient, tumor resection was successful in reducing her renin back to normal values within 3 weeks. Unfortunately, our patient's case highlights the risk of organ damage with chronic hypertension. This patient showed signs of arteriole remodeling (Figure 5) and was found to have Stage III chronic kidney disease from the months to possible years of undiagnosed renin-mediated hypertension. Our case follows reports of resolution of hypertension in 89% of patients and normalization of renin values in 95% of patients after tumor resection (both complete nephrectomy and partial nephrectomy have been utilized successfully). However, patients with JGCT have shown complications including cardiopathy in 42% (including 5% congestive heart failure), nephropathy in 32% (including 7.7% renal dysfunction or failure), and retinopathy 40% that can be irreversible [9].

While this tumor is generally considered benign, cases with malignant potential, metastatic lesions, and vascular invasion have been described including cases in children [5–7, 11, 12]. What is interesting and important to note is that we found this to be a PET avid tumor. PET imaging is frequently used in staging pediatric tumors. Obviously, concerns for malignancy heavily influence the decision to proceed with biopsy versus partial resection (something strongly contraindicated in most malignant renal tumors) versus complete nephrectomy. To the authors' knowledge, less than a handful of case reports have evaluated PET avidity. All have found JGCT tumors to be PET avid, even one case where the JGCT was no longer functioning [7, 13, 14]. Thus, physicians should take pause in cases of PET avid renal tumors with simultaneous significant hypertension as that may represent this very rare benign disease. JGCTs have also been reported to result in elevated erythropoietin levels with erythrocytosis [15], a feature not observed in our patient.

In summary, JGCT is a benign tumor affecting mostly adolescent females. It is a curable cause of hypertension and should be included in the differential diagnosis of suspected renin-mediated hypertension as the delay in diagnosis might lead to irreversible renal tissue injury with subsequent progressive kidney disease, and suspicion for the diagnosis could result renal sparing surgery.

## Data Availability

Data sharing is not applicable to this article as no datasets were generated or analyzed during the current study.

## References

[1] Robertson P. W., Klidjian A., Harding L. K., Walters G., Lee M. R., Robb-Smith A. H. (1967). Hypertension Due to a Renin-Secreting Renal Tumour. *The American Journal of Medicine*.

[2] Inam R., Gandhi J., Joshi G., Smith N. L., Khan S. A. (2019). Juxtaglomerular Cell Tumor: Reviewing a Cryptic Cause of Surgically Correctable Hypertension. *Current Urology*.

[3] Geisler D., Almutairi F., John I. (2022). Malignant Juxtaglomerular Cell Tumor. *Urology Case Reports*.

[4] Sakiyama H., Hamada S., Oshiro T. (2023). Juxtaglomerular Cell Tumor with Pulmonary Metastases: A Case Report and Review of the Literature. *Pediatric Blood and Cancer*.

[5] Capovilla M., Couturier J., Molinie V. (2008). Loss of Chromosomes 9 and 11 May Be Recurrent Chromosome Imbalances in Juxtaglomerular Cell Tumors. *Human Pathology*.

[6] Duan X., Bruneval P., Hammadeh R. (2004). Metastatic Juxtaglomerular Cell Tumor in a 52-Year-Old Man. *The American Journal of Surgical Pathology*.

[7] Daniele A., Sabbadin C., Costa G. (2018). A 10-year History of Secondary Hypertension: a Challenging Case of Renin-Secreting Juxtaglomerular Cell Tumor. *Journal of Hypertension*.

[8] McVicar M., Carman C., Chandra M., Abbi R. J., Teichberg S., Kahn E. (1993). Hypertension Secondary to Renin-Secreting Juxtaglomerular Cell Tumor: Case Report and Review of 38 Cases. *Pediatric Nephrology*.

[9] Dong H., Zuo Y., An X. (2024). Clinical Features, Laboratory Findings and Treatment of Juxtaglomerular Cell Tumors: a Systemic Review. *Hypertension Research*.

[10] Tullus K., Brennan E., Hamilton G. (2008). Renovascular Hypertension in Children. *The Lancet*.

[11] Niikura S., Komatsu K., Uchibayashi T. (2000). Juxtaglomerular Cell Tumor of the Kidney Treated with Nephron-Sparing Surgery. *Urologia Internationalis*.

[12] Haab F., Duclos J. M., Guyenne T. T., Plouin P. F. (1995). Reninsecreting Tumors: Diagnosis, Conservative Surgical Approach and Long-Term Results. *The Journal of Urology*.

[13] Jiang Y., Hou G., Zhu Z., Zang J., Cheng W. (2020). Increased FDG Uptake on Juxtaglomerular Cell Tumor in the Left Kidney Mimicking Malignancy. *Clinical Nuclear Medicine*.

[14] Dong J., Xu W., Ji Z. (2020). Case Report: A Nonfunctioning Juxtaglomerular Cell Tumor Mimicking Renal Cell Carcinoma. *Medicine (Baltimore)*.

[15] Remynse L. C., Begun F. P., Jacobs S. C., Lawson R. K. (1989). Juxtaglomerular Cell Tumor with Elevation of Serum Erythropoietin. *The Journal of Urology*.

